# Polymyositis as a paraneoplastic syndrome of a patient with primary pulmonary lymphoepithelioma-like carcinoma: a case report and literature review

**DOI:** 10.1186/s13019-022-01860-4

**Published:** 2022-05-17

**Authors:** Yu Lei, Caiyang Liu, Xiu Wan, Yanhui Yang, Yi Yao, Lei Luo, Tingyu Huang, Ji Li

**Affiliations:** 1 Department of Cardiothoracic Surgery, The First People’s Hospital of Neijiang, Shizhong District, No. 1866, West Section of Hanan Avenue, Neijiang, 641000 Sichuan China; 2 Department of Pathology, The First People’s Hospital of Neijiang, Neijiang, 641000 Sichuan China

**Keywords:** Pulmonary lymphoepithelioma-like carcinoma, Lung cancer, Polymyositis, Paraneoplastic syndrome, Treatment, Prognosis

## Abstract

**Background:**

Pulmonary lymphoepithelioma-like carcinoma (LELC) is a rare type of non-small cell lung cancer, which mostly occurred in non-smoking Asian populations. The prognosis of this tumor is better than other lung cancers. Polymyositis, a kind of idiopathic inflammatory myopathies, may negatively affect the prognosis of patients with lung cancer as a paraneoplastic syndrome (PNPS). LELC is seldomly accompanied by PNPS, thus the treatment strategy and prognosis should be discussed.

**Case presentation:**

We report a 49-year-old female patient who was hospitalized for “symmetric limb weakness and pain for more than 2 months”. Glucocorticoid-based anti-inflammatory therapy had been performed for over 3 weeks before the patient was hospitalized, however, in vain. The result of serum autoimmune antibody showed Anti-nRNP/Sm ( +). The serum level of myoglobin, lactate dehydrogenase and creatine kinase elevated significantly. An electromyogram revealed peripheral nerves injury and myogenic damages. Imaging showed a mass in the posterior basal segment of the left lung. A percutaneous transthoracic needle biopsy was performed and the pathological result was LELC. The patient was diagnosed with pulmonary LELC accompanied by polymyositis. Positron emission tomography-computed tomography (PET-CT) showed only ipsilateral hilar and mediastinal lymph nodes metastasis. Video-assisted thoracoscopic left lower lobectomy and systematic mediastinal lymphadenectomy were performed. The postoperative pathological stage was T2N2M0, IIIA (UICC 8th), and the patient received adjuvant chemotherapy and subsequent radiotherapy. The patient was followed up for 5 months with no recurrence of tumor and the limb weakness and pain were relieved apparently after the successful comprehensive treatment of her primary tumor.

**Conclusion:**

Pulmonary LELC is a rare subtype of non-small cell lung cancer seldomly accompanied by PNPS. Though polymyositis is associated with lung cancer, it is easy to ignore this relationship when a patient is diagnosed with LELC in the clinic. Surgery based comprehensive treatment of primary tumor can lead to a prospective prognosis in pulmonary LELC patients with PNPS. And successful treatment of pulmonary LELC can also improve symptoms of PNPS.

## Introduction

Pulmonary lymphoepithelioma-like carcinoma (LELC) is a rare kind of non-small cell lung cancer (NSCLC) that is similar to undifferentiated nasopharyngeal carcinoma in morphology histologically [1]. The Epstein–Barr Virus (EBV) infection was proven to have an association with the pathogenesis of this disease [2]. Manifestations of pulmonary LELC are similar to other lung cancers. However, intrabronchial involvement is rarely detected so that irritable cough and hemoptysis seldomly happens [3]. Besides, it is difficult to differentiate pulmonary LELC from other lung cancers on imaging [4, 5]. Microscopically, the phenomenon that intense lymphocytic and plasma cell infiltration in the stroma and intermixed with the tumor cells can distinguish pulmonary LELC from other lung cancers [6]. Polymyositis (PM), which is defined as an inflammatory myositis with no rash according to Bohan and Peter’s criteria, is thought to be associated with cancer [7, 8]. A literature review on lung cancer accompanied with PM showed that small cell carcinoma, squamous cell carcinoma and adenocarcinoma were the most common pathological subtypes [9]. However, there is still no report of pulmonary LELC accompanied by PM. The pathogenesis of PM is still unclarified, but the therapeutic schedule is unified around the world. Glucocorticoids are used as the first-line treatment despite several adverse effects [10]. More specifically, the treatment of cancer usually leads to an improvement in the PM-related symptoms among the patients suffering from cancer accompanied by PM [11].

## Case report

**Fig. 1:**
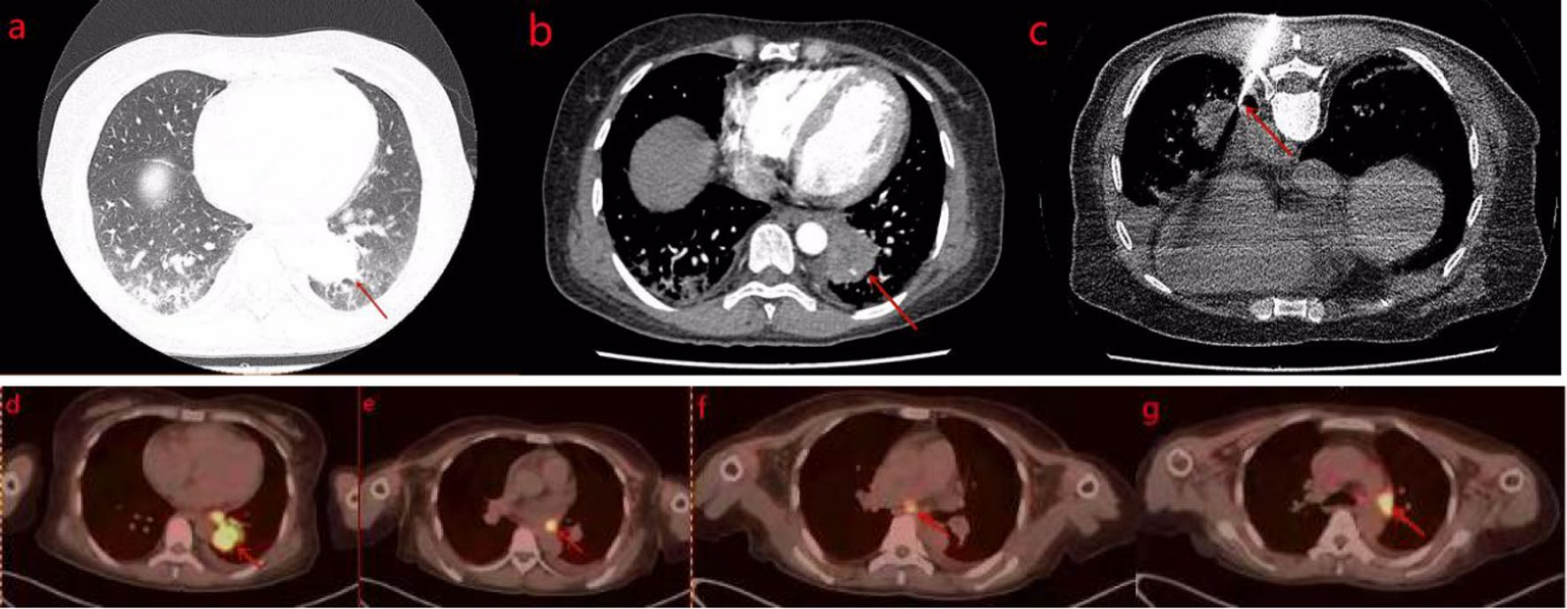
**a** Chest CT revealed a mass in the posterior basal segment of the left lower lobe (size 3.7 × 3.3 cm) with poorly-defined boundary, peripheral burr and lobulation sign. **b** Enhanced CT showed inhomogeneous enhancement of the mass with the branches of left inferior pulmonary artery and vein passing through. **c** CT-guided percutaneous transthoracic biopsy. **d**–**g** PET/CT showed SUV_max_ of the mass, the lpsilateral hilar and mediastinal lymph nodes were high

**Fig. 2:**
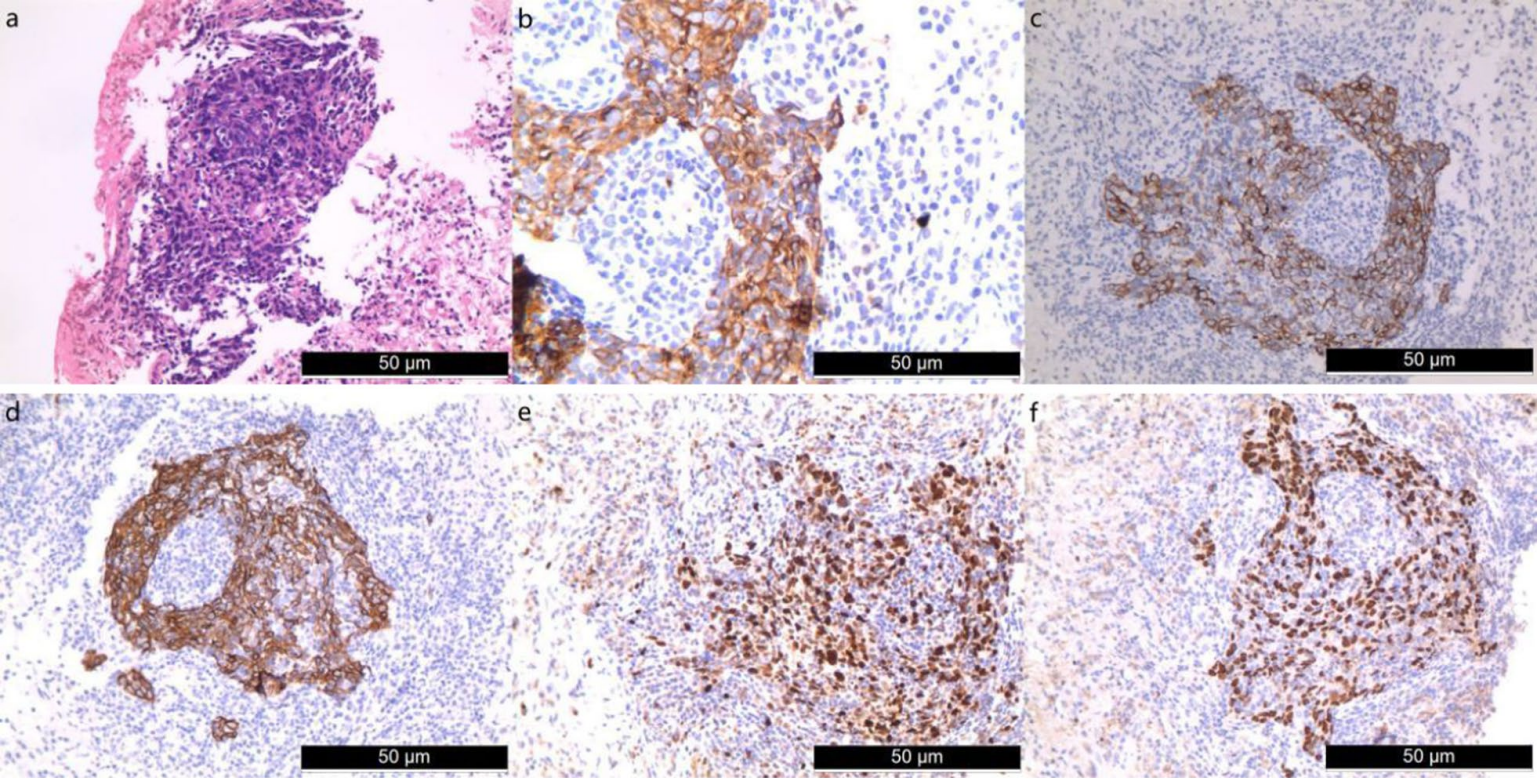
**a** Hematoxylin and eosin staining showed neoplastic cells were with irregular nuclei and epithelioid appearance,massive lymphocytic infiltration could be found in the stroma. Immunohistochemistry demonstrated **b** CK (+), **c** CK19 (+), **d** CK56 (+), **e** Ki-67 (+, index of approximately 50%) and **f** p63(+). Original magnification × 200

The patient was a 49-year-old Chinese woman without a history of smoking or medication, who developed symmetric limb weakness and pain for more than 2 months. The patient visited a local hospital and been performed some imaging examinations on her knees, shoulder joint and lumbar vertebra without any significant result. Autoimmune diseases were suspected and glucocorticoid-based treatment was performed for more than 3 weeks with no improvement of her symptoms observed. The patient visited our outpatient department for further treatment in December 2020, during which period every patient needed performed chest computed tomography (CT) due to the epidemic of COVID-19. The CT scan revealed a mass in the posterior basal segment of the left lower lobe (size, 3.7 × 3.3 cm) with poorly-defined boundary, peripheral burr and lobulation sign. Slight infection of bilateral lower lobes and enlargement of hilar and mediastinal lymph nodes were also observed (Fig. 1a). The patient was admitted to the department of thoracic surgery of the First People’s Hospital of Neijiang on December 23, 2020. Neurological physical examination revealed slight muscle strength weakness. The pectoralis major reflex, Hoffmann’s sign, ankle clonus and tendon reflex were all positive. The result of the electromyogram (EMG) revealed peripheral nerve injury in limbs, nerve root injury and myogenic lesion. The enhanced chest CT showed inhomogeneous enhancement of the mass with the branches of the left inferior pulmonary artery and vein passing through (Fig. 1b). The fiberoptic bronchoscopy was performed without any positive finding of neoplasm or cast-off cells. The significant laboratory measurements were as follow: myoglobin (MB) 433.8 ng/mL (reference value: 6.3–70.9 ng/mL), creatine kinase 1325U/L (reference value: 40–200 U/L), lactate dehydrogenase (LDH) 573U/L (reference value: 100-350U/L), erythrocyte sedimentation rate 34 mmol/L (reference value: 0-20 mm/h), Aspartate aminotransferase (AST) 79U/L (reference value: 8-20U/L), alanine aminotransferase (ALT) 42U/L (reference value: 0-40U/L). Autoimmune antibody tests showed only Anti-nRNP/Sm was positive (> 400RU/ml). After a multi-disciplinary consultation, the CT-guided percutaneous transthoracic biopsy was suggested to be performed (Fig. 1c). The pathological report showed neoplastic cells were with irregular nuclei and epithelioid appearance, and massive lymphocytic infiltration could be found in the stroma. The immunohistochemistry results were as follow: AE1/AE3 (+), LCA (–), Ki-67 (+, 50%), HMB45 (–), P63 (+), Vimentin (–), TdT (–), S-100 (–), CD21 (–), CD1a (–), CD163 (+, histocyte), EMA (+), CK5/6 (+), CK19 (+), Langerin (–) (Fig. 2). Epstein–Barr encoding region (EBER) in situ hybridization (ISH) was not performed due to the constraint of the laboratory. Taking into account micromorphology and immunohistochemistry, LELC was diagnosed. The fluorine-18 fluorodeoxyglucose PET-CT (^18^F-FDG PET-CT) was performed which revealed the maximum standard uptake values (SUV_max_) of the mass, the ipsilateral hilar and mediastinal lymph nodes were high (Fig. 1d–g). The SUV_max_ of other organs including the nasopharynx didn’t elevate.

**Fig. 3:**
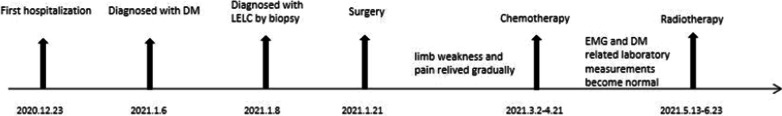
Treatment timeline

According to the patient’s history, clinical manifestations and examination results, she was diagnosed with pulmonary LELC (cT2N2M0, IIIA) accompanied by PM. Video-assisted thoracoscopic left lower lobectomy and systematic mediastinal lymphadenectomy were performed. The postoperative pathology result was the same as the needle biopsy tissue. In addition, perineural invasion but no visceral pleural invasion was observed. Metastases were found in five lymph nodes (5/14), which included level 4 (2/2), level 5 (0/2), level 6 (0/3), level 7 (1/1), level 9 (2/2), level 10 (0/1), level 11 (0/1), level 12 (0/2). The postoperative pathological stage was pT2N2M0, IIIA (UICC 8th), so she received adjuvant chemotherapy (5-fluorouracil combined with Cisplatin) 3 times (5-fluorouracil 1.125 g d1-4, Cisplatin 35 mg d5-7) and subsequent radiotherapy (PCTV 5040 cGy PGTVnd 5600 cGy). She was followed up for 5 months with no recurrence of tumor. And the symptom of symmetric limb weakness and pain relieved a lot due to the successful comprehensive treatment of LELC. The serum values of creatine kinase, Mb and Anti-nRNP/Sm also decreased gradually (creatine kinase 209U/L, MB 198U/L, Anti-nRNP/Sm 147.47RU/mL 1 month after surgery. All the laboratory indicators above became normal after radiotherapy). The reexamined result of EMG 1 month after surgery revealed very slight peripheral nerves injury and atypical myogenic damage, which meant a good improvement of the PM. The treatment timeline was as follows (Fig. 3).

## Discussion

LELC, originally described in the nasopharynx, refers to undifferentiated carcinoma with predominant lymphocytic infiltration [12]. Zhu et al. [13] reported that LELC mostly originates from organs of the fore-gut. Recently, there are several reports describing some cases that suffer from LELC of other organs such as the rectum, prostate and colon [14–16]. It was first reported originating from the lung in 1987 by Begin as a large-cell carcinoma [17]. Later, pulmonary LELC was found to be similar to poorly differentiated squamous cell carcinoma [18]. In 2015, the World Health Organization’s histological classification of lung tumors classified pulmonary LELC as an untyped tumor [19].

Primary pulmonary LELC has been proven to be strongly associated with EBV infection in Asians by detecting high levels of antibodies against EBV-capsid antigens in the patient’s serum [17]. The association was also observed in some specific geographic groups including Chinese, Japanese, and Eskimos later [20]. However, the situation seemed to be different in western patients. In Claudia’s report, the EBV genome was not detected through ISH in any of the patients’ serum with pulmonary LELC from western countries [2]. Maybe the etiology of pulmonary LELC differs among races and geography. Primary pulmonary LELC occurs mostly in young Asians [21]. Otherwise, the median age at diagnosis of the western patients is 65 [22]. Compared to other lung cancers, smoking does not affect the morbidity of LELC that apparently [21]. Furthermore, there seem to be no differences between sexes [22].

To diagnose primary pulmonary LELC, a chest CT scan should be the first chosen examination. Ma et al. [23] concluded that the CT scan features of pulmonary LELC usually appeared as a large, central, well defined and lobulated mass and enhanced CT usually showed inhomogeneous or homogeneous enhancement of the mass with vascular or bronchial encasement. PET imaging is wildly used to investigate the malignant potential of solitary pulmonary nodules which is more than 8 mm in diameter [24]. LELC is an 18F-FDG-avid tumour, PET imaging can provide valuable information on the disease detection, staging and treatment response evaluation [25]. The role that serum tumor markers play in the diagnosis of pulmonary LELC is depressing. Ying et al. [26] reported neuron-specific enolase and cytokeratin 19 fragment 21–1 elevated in half of their cases, but they still lack of adequate proofs to certify the association between tumor markers and pulmonary LELC. In our case, the serum levels of tumor markers were normal at the time pulmonary LELC was diagnosed. Roger et al. [27] reported free circulating serum EBV-DNA could be detected in most patients with untreated or relapsed pulmonary LELC. However, it’s impractical to use serum EBV-DNA as a tumor marker for diagnosis, because pulmonary LELC is a relatively rare subtype of lung cancer in the clinic. Perhaps serum EBV-DNA can be used to monitor therapy response or relapse surveillance of pulmonary LELC.

There is no unified therapeutic schedule for pulmonary LELC, and most studies published about this disease follow the National Comprehensive Cancer Network guidelines of NSCLC. Complete resection is the first choice for patients with pulmonary LELC in the early stage (stage I and stage II) [22]. Although pulmonary LELC is sensitive to chemotherapy due to the similar histological and biological characteristics to nasopharyngeal carcinoma, adjuvant chemotherapy doesn’t improve the postoperative overall survival (OS) of patients with early- stage disease [28, 29]. However, adjuvant chemotherapy has been identified to significantly improve the prognosis for patients at stage IIIA who received complete resection [26, 28]. As for the patients at the advanced stage, chemotherapy can get a good treatment response. It’s reported that platinum-based doublets chemotherapy for the advanced stage patients could prolong the OS as well as radical surgery to the early- stage patients under a 67 month median follow-up duration [22]. If radiotherapy can improve the prognosis of pulmonary LELC is still uncertain. Qi et al. [30] recently published a retrospective analysis result including 922 non-nasopharyngeal LELC patients indicating no significant improvement of cancer-specific survival was observed intervened by radiotherapy. However, this research didn’t uniquely analyze pulmonary LELC cases, and the diversity among different stages wasn’t discussed forward either. Target therapy in pulmonary LELC seems to be unpromising. Epidermal growth factor receptor mutation and anaplastic lymphoma kinase rearrangement were demonstrated uncommon in pulmonary LELC in previous research [31, 32]. Immunotherapy may have a good prospect in treating pulmonary LELC according to recent studies. Chang et al. [33] detected PD-L1 expression in 66 patients with pulmonary LELC and the positive rate (defined as > 5%) was 75.8%. And Wu et al. [34] reported high level of PD-L1 expression (defined as ≥ 50%) was found in 61% (36/59) of patients. A recent study compared the therapeutic effect of immunotherapy with chemotherapy in advanced-stage patients, and longer progression-free survival (PFS) was achieved in the former group [35]. Further large sample clinic studies of immunotherapy pulmonary LELC are needed. As for prognosis, most former studies demonstrated that of pulmonary LELC was better than other NSCLCs. He et al. [22] reported that the OS rate by 1, 3 and 5 years of patients with pulmonary LELC could reach 85.6%, 68.9% and 59.5%, compared to 39.1%, 18% and 12.9% of other NSCLCs.

Idiopathic inflammatory myopathies (IIM), collectively known as myositis, are heterogeneous disorders characterized by muscle weakness and muscle inflammation [36]. PM, which is one of the most common subgroups of IIM, should be suspected in any patient who presents with progressive, varying degrees of symmetric proximal limb and truncal muscle weakness. As for myositis-specific antibodies, serum Anti-Jo-1 autoantibody is usually detected positive in patients with IIM [37]. In our case, however, Anti-nRNP/Sm is the only positive finding. Other laboratory measurements such as the serum levels of creatine kinase, LDH, ALT and AST usually elevate just the same as in our case [38]. The patient in our case is diagnosed with PM due to her age, muscle weakness, laboratory measurements according to the 2017 European League Against Rheumatism/ American College of Rheumatology classification criteria for adult and juvenile idiopathic inflammatory myopathies and their major subgroups [39]. Therapeutically, glucocorticoids are used as the first-line treatment, and concomitant treatment with steroid-sparing immunosuppressive agents can reduce the relapse risk during glucocorticoid tapering and the initial doses of glucocorticoids [40, 41]. However, the muscle weakness and pain of the patient didn’t relieve at all after more than 20 days of glucocorticoid therapy. Thus, the traditional drug therapy may make no sense when PM is accompanied by cancer.

Catherine et al. [8] the reported PM was associated with an increased risk of lung cancer. On the other hand, autoimmune disease as paraneoplastic syndrome (PNPS) is not rare in lung cancer, and the incidence rate is about 4.7% [42]. Thus, when a patient visits outpatient suspected of IIM, a chest CT scan should be performed routinely. In our case, the diagnosis and treatment of the patient were delayed to a certain extent due to the unawareness of the correlation between lung cancer and PM. Previous studies showed that PNPS among patients with SCLC was higher than NSCLC cause all SCLC were derived from neuroendocrine cells which could secrete peptide hormones [43]. Pulmonary LELC is a kind of NSCLC and belongs to squamous cell lineage immunohistochemically [1]. Till now, there’s no research has found particular peptides, hormones or cytokines secreted by LELC tumor cells that may lead to PNPS. And here are only two case reports about pulmonary LELC with PNPS that can be found. Zhu et al. [13] reported a patient diagnosed with hypertrophic pulmonary osteoarthropathy (HPOA) by emission computed tomography (ECT) which is considered to be PNPS of pulmonary LELC. Though the relief of HPOA couldn’t be evaluated because the patient refused to be re-examined by ECT after surgery for the primary tumor. The other report was about a patient diagnosed at IIIB clinical-stage accompanied with erythema elevatum diutinum (EED). The skin lesions didn’t fade at all after topical steroid treatment. Combination of chemotherapy and radiotherapy achieved over 3 years of disease-free survival and marked relief of EED [44]. We concluded some data of the two cases in Table 1. Review our case, the patient’s muscle weakness and pain were relieved gradually during the comprehensive therapy of her tumor. The PM-related laboratory measurements such as creatine kinase, MB and Anti-nRNP/Sm also decreased obviously when re-examined after radiotherapy. And the postoperative EMG showed minimal peripheral nerves injured and atypical myogenic damage which meant her muscular damage was relieved a lot. Here we can see, it’s the primary tumor should be treated firstly rather than the PNPS caused by it. Though Dumansky et al. [42] concluded PNPS negatively affected the survival rates of patients with lung cancer, the prognosis of pulmonary LELC patients with PNPS seemed prospective.

**Table 1:** Summary of two case reports about pulmonary LELC accompanied with PNPS

Source	Age (y)	Gender	TS (cm)	Location	LNM	DM	EBER	Surgery	CT	RT	PNPS	PI
Zhu et al.	49	M	3.1 × 3.5/	LLL	No	No	+	Yes	No	No	HOPA	UK
			1.8 × 2.0									
Liu et al.	46	F	UK	RML	UK	UK	+	No	Yes	Yes	EED	Yes

M, male; F, female; TS, tumor size; LLL, Left lower lobe; RML, Right middle lobe; LNM, lymph node metastasis; DM, distant metastasis; CT, chemotherapy; RT, radiotherapy; + , positive; PI, PNPS improvement; UK, unknown

## Conclusion

PM is associated with lung cancer, ignoring this relationship may lead to missed diagnosis in the clinic. Pulmonary LELC is a rare subtype of NSCLC seldomly accompanied with PNPS. Though PNPS negatively affected the prognosis of patients with other lung cancers, surgery based comprehensive treatment of primary tumor can lead to a prospective prognosis in pulmonary LELC patients with PNPS. And successful treatment of pulmonary LELC can also improve symptoms of PNPS.

## Data Availability

Not applicable.

## Abbreviations

LELC: Lymphoepithelioma-like carcinoma
PNPS: Paraneoplastic syndrome
NSCLC: Non-small cell lung cancer
EBV: Epstein–Barr Virus
PM: Polymyositis
CT: Computed tomography
PET- CT: Positron emission tomography-computed tomography
EMG: Electromyogram
MB: Myoglobin
LDH: Lactate dehydrogenase
AST: Aspartate laminotransferase
ALT: Alanine aminotransferase
EBER: Epstein–Barr encoding region
ISH: In situ hybridization
OS: Overall survival
IIM: Idiopathic inflammatory myopathies
HPOA: Hypertrophic pulmonary osteoarthropathy
ECT: Emission computed tomography
EED: Erythema elevatum diutinum
